# Ferromagnetic behaviour of Fe-doped ZnO nanograined films

**DOI:** 10.3762/bjnano.4.42

**Published:** 2013-06-13

**Authors:** Boris B Straumal, Svetlana G Protasova, Andrei A Mazilkin, Thomas Tietze, Eberhard Goering, Gisela Schütz, Petr B Straumal, Brigitte Baretzky

**Affiliations:** 1 Karlsruher Institut für Technologie, Institut für Nanotechnologie, Hermann-von-Helmholtz-Platz 1, 76344 Eggenstein-Leopoldshafen, Germany; 2 Institute of Solid State Physics, Russian Academy of Sciences, Ac. Ossipyan str. 2, 142432 Chernogolovka, Russia; 3 Max-Planck-Institut für Intelligente Systeme, Heisenbergstrasse 3, 70569 Stuttgart, Germany; 4 National University for Research and Technology “MISiS”, Leninsky prospect 4, 119991 Moscow, Russia; 5 Institute of Metallurgy and Materials Science, Russian Academy of Sciences, Leninsky prospect 49, 117991 Moscow, Russia

**Keywords:** Fe, ferromagnetism, grain boundaries, ZnO

## Abstract

The influence of the grain boundary (GB) specific area *s*_GB_ on the appearance of ferromagnetism in Fe-doped ZnO has been analysed. A review of numerous research contributions from the literature on the origin of the ferromagnetic behaviour of Fe-doped ZnO is given. An empirical correlation has been found that the value of the specific grain boundary area *s*_GB_ is the main factor controlling such behaviour. The Fe-doped ZnO becomes ferromagnetic only if it contains enough GBs, i.e., if *s*_GB_ is higher than a certain threshold value *s*_th_ = 5 × 10^4^ m^2^/m^3^. It corresponds to the effective grain size of about 40 μm assuming a full, dense material and equiaxial grains. Magnetic properties of ZnO dense nanograined thin films doped with iron (0 to 40 atom %) have been investigated. The films were deposited by using the wet chemistry “liquid ceramics” method. The samples demonstrate ferromagnetic behaviour with *J*_s_ up to 0.10 emu/g (0.025 μ_B_/f.u.ZnO) and coercivity *H*_c_ ≈ 0.03 T. Saturation magnetisation depends nonmonotonically on the Fe concentration. The dependence on Fe content can be explained by the changes in the structure and contiguity of a ferromagnetic “grain boundary foam” responsible for the magnetic properties of pure and doped ZnO.

## Introduction

The possibility of ferromagnetism (FM) in oxides has been widely debated since 2000. In their theoretical work, Dietl et al. discussed the chances for oxides to possess saturation of magnetisation in an external magnetic field, coercivity, and a Curie temperature above room temperature (RT) [1]. According to their theory, FM could appear if one dopes the oxides (especially ZnO) with “magnetic” atoms such as Co, Mn, or Fe. Such transition-metal (TM) ions induce FM ordering into a magnetically polarized oxide lattice modified by doping. After publication of their paper [1] a lot of theoretical and experimental works were carried out in order to find the “promised” FM behaviour of zinc oxide (see [2–6] and references therein). However, the obtained results were quite contradictory. Several teams of experimentalists reported observations of weak but quite reproducible ferromagnetism. Other research groups never succeeded in synthesizing ferromagnetic ZnO. The huge interest in FM in ZnO is because it is a cheap semiconductor that is widely used in various devices and technologies. The FM behaviour, in addition to the attractive optical and semiconductor properties, could open the way for the future applications of FM ZnO in spintronics [2]. Recently we proposed an explanation for the contradictory results in the investigations of FM ZnO [6]. We observed, that FM behaviour does not appear in bulk ZnO (even doped by Mn or Co), but only in polycrystalline samples with very high specific area *s*_GB_ of grain boundaries (GBs), i.e., the ratio of GB area to grain volume [6]. Only in the case where the specific area of grain boundaries in ZnO exceeds a certain threshold called *s*_th_, does the ferromagnetism appear. If *s*_GB_ is high enough, even the doping by TM ions is not essential, and FM appears in pure, undoped ZnO. The viewpoint that GBs are the reason for FM in ZnO became generally accepted in the past few years [3,7–16]. Therefore, it is important to continue the investigations into the GB-induced ferromagnetic behaviour of TM-doped ZnO. We were able to observe the FM behaviour even in pure ZnO due to the extremely small grain size in our films deposited by the original method of so-called “liquid ceramics”, which is based on the application of organic acids for the solution of metallic ions for pure and Mn- and Co-doped ZnO [6,17–18].

The observed dependence of the saturation magnetization of Mn- and Co-doped ZnO on the Mn and Co concentration showed complicated nonmonotonic behaviour [17–18]. The concentration dependence for Co-doped ZnO films has one maximum [18], and the concentration dependence for Mn-doped ZnO films has two maxima [17]. The shape of the dependence of the saturation magnetization on the Mn and Co concentration is different for the Mn- and Co-doped nanograined ZnO manufactured by different methods. It is most probably controlled by the topology of the GB network (ferromagnetic GB foam) in the ZnO polycrystals. Our findings strongly suggest that GBs and related vacancies are the intrinsic origin of RT ferromagnetism. We can also suppose another reason for the fact that the concentration dependence of the saturation magnetization for Co-doped ZnO films has one maximum [18], and the concentration dependence for Mn-doped ZnO films has two maxima [17]. It is probably due to the fact that cobalt demonstrates only one oxidation state Co^3+^ whereas manganese can possess several oxidation states, namely +2, +3 and +4 [17–18]. Together with cobalt and manganese, iron is one of the most important dopants in ZnO. Similar to manganese, iron has different oxidation states (Fe^2+^ and Fe^3+^). This fact prompts us to check, whether the concentration dependence of the saturation magnetization for Fe-doped ZnO films has one or two maxima. Therefore, the goals of this work are to determine the threshold value *s*_th_ of the specific GB area for Fe-doped zinc oxide and to analyse experimentally the influence of Fe on the saturation magnetization of ZnO in a broad interval of Fe concentrations.

## Experimental

Pure and Fe-doped ZnO thin films consisting of dense equiaxial nanograins were produced by using the novel method of liquid ceramics [19]. Zinc(II) butanoate diluted in an organic solvent with zinc concentrations between 1 and 4 kg/m^3^ was used as a precursor for the preparation of pure ZnO films. For the ZnO films that were doped with 0.1, 5, 12, 20, 31, and 40 atom % Fe, zinc(II) butanoate solution was mixed with an iron(III) butanoate solution in suitable proportions. The butanoate precursor was deposited onto (102) single-crystalline sapphire substrates. Drying at 100 °C in air for about 30 min was followed by thermal pyrolysis in an electrical furnace in air at 550 °C. The Zn and Fe content in doped oxides was measured by atomic absorption spectroscopy in a Perkin-Elmer spectrometer and electron-probe microanalysis (EPMA). EPMA investigations were carried out in a Tescan Vega TS5130 MM microscope equipped by the LINK energy-dispersive spectrometer produced by Oxford Instruments. The presence of other magnetic impurities, such as Mn, Co, and Ni, was below 0.001 atom %. During the long preparation procedure all possible precautions were taken to exclude any additional FM contaminations (for example, nonmagnetic ceramic scissors and tweezers, etc., were used). It is known from the literature [20] that the effect of a contaminated substrate can completely conceal the ferromagnetic signal of ZnO itself. We carefully measured the magnetization curves for bare Al_2_O_3_ substrates and subtracted them from data for the substrates including ZnO films. The films were transparent and sometimes with a very slight greenish finish. The films had a thickness between 50 and 200 nm, determined using edge-on transmission electron microscopy (TEM) and EPMA. TEM investigations were carried out on a Jeol JEM–4000FX microscope at an accelerating voltage of 400 kV. X-ray diffraction (XRD) data were obtained on a Siemens diffractometer (Cu Kα radiation). Evaluation of the grain size *D* from the X-ray peak broadening was performed by using the Scherrer equation [21]. The magnetic properties were measured on a superconducting quantum interference device (Quantum Design MPMS-7 and MPMS-XL). The magnetic field was applied parallel to the sample plane (“in plane”). The diamagnetic background signals, generated by the sample holder and the substrate, were carefully subtracted, due to the small absolute magnetic moments measured in the range of 10^−6^ to 10^−4^ emu.

## Results

**Figure 1:**
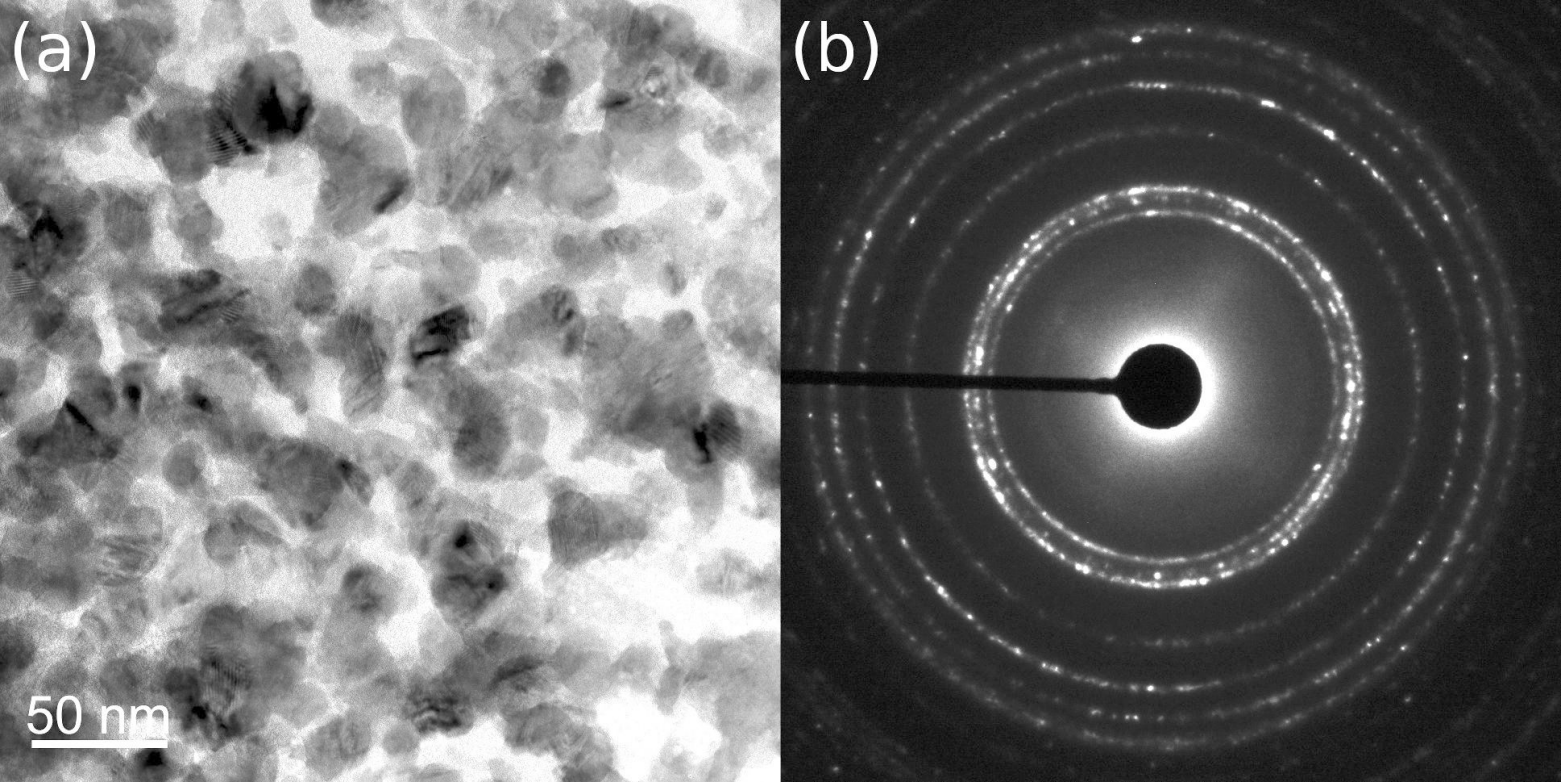
(a) Bright-field TEM micrograph of the nanograined pure ZnO thin film deposited on a sapphire substrate by the novel liquid ceramics method. Electron diffraction pattern (b) shows only rings from the ZnO wurtzite structure; no texture is visible. Bright spots originate from the sapphire substrate.

Using the method of liquid ceramics, we deposited nanograined (the size of equiaxial grains was 10 to 30 nm) and poreless pure and Fe-doped ZnO thin films (see micrographs in Figure 1a). In the samples with 0.1, 5, 12, and 20 atom % Fe only pure quartzite grains are present, according to the studies with selected area diffraction (Figure 1b), TEM and XRD. These methods reveal the presence of ternary cubic zinc–iron oxide ZnFe_2_O_4_ in samples with 31 and 40 atom % Fe. No visible texture can be observed in the deposited thin films, namely the diffraction rings shown in Figure 1b are uniform without any preferred orientations of ZnO grains.

**Figure 2:**
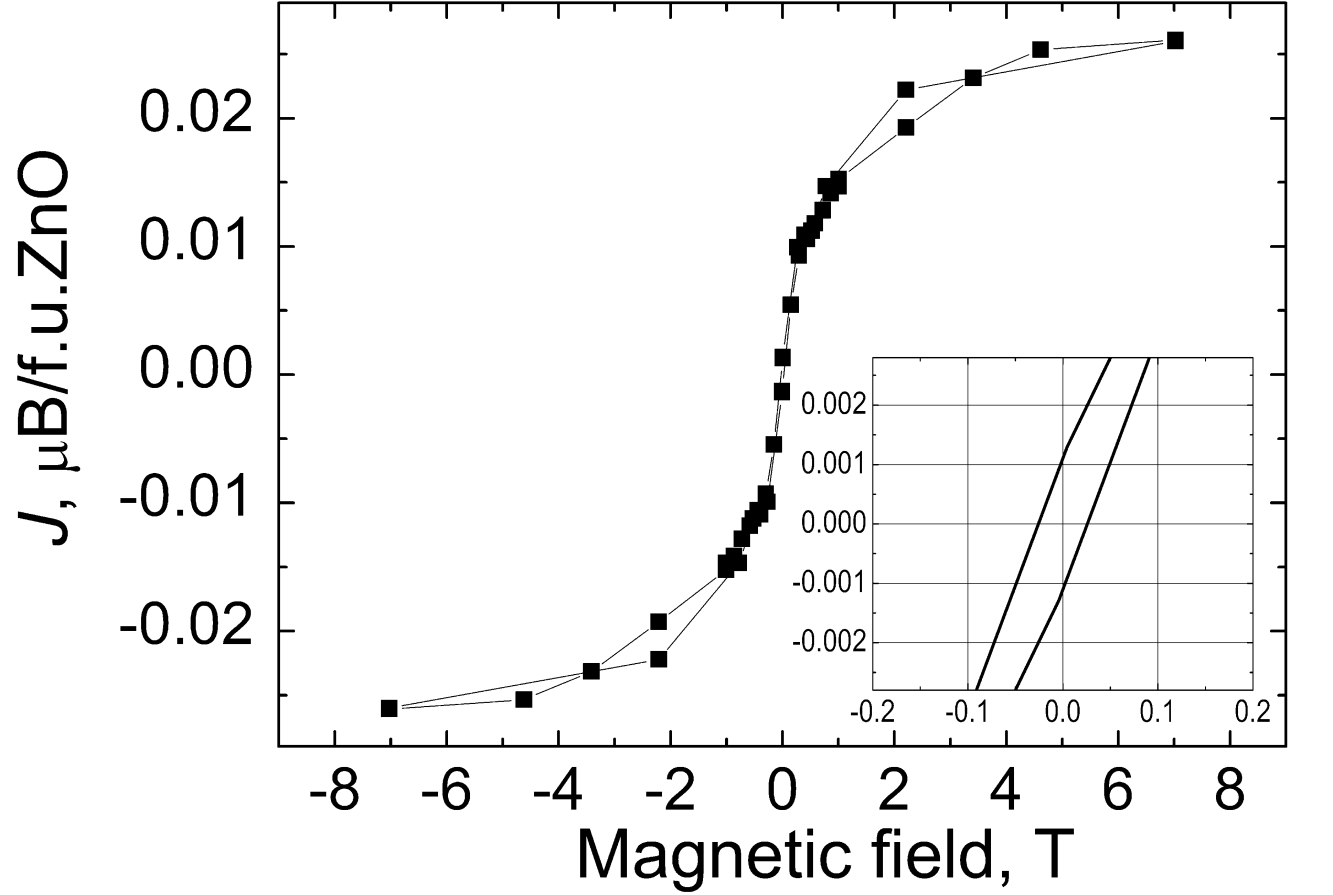
Magnetization (calibrated in units of Bohr magnetons per formula unit of ZnO) at RT for ZnO thin films doped with 0.1 atom % Fe deposited on the sapphire substrate. The curve was obtained after subtracting the magnetic contribution from the substrate and the sample holder. The inset shows the magnified central part of the magnetisation curve.

The observed FM behaviour in doped nanocrystalline as well as in dense ZnO films with 0.1 atom % Fe is depicted in Figure 2. Shown is the pronounced FM indicated by the saturation of magnetization (*J*_s_ ≈ 0.10 emu/g or 0.025 μ_B_/f.u.ZnO (units of Bohr magnetons per formula unit of ZnO) above the applied field ≈1.5 T) and hysteretic behaviour with coercivity *H*_c_ ≈ 0.03 T (see the inset). These magnetization and coercivity values are close to those obtained by other methods for the Fe-doped samples [22–47].

**Figure 3:**
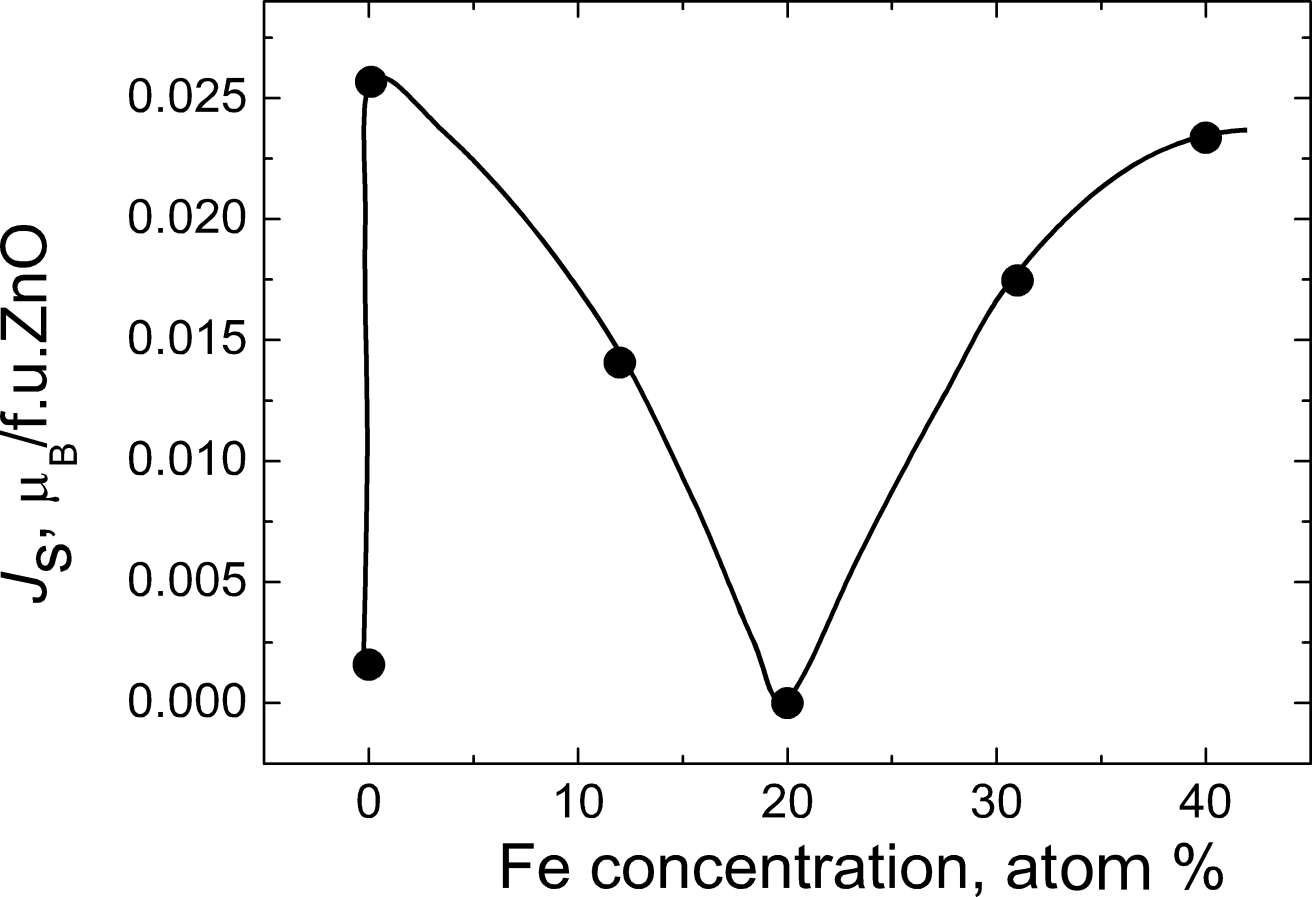
Dependence of the saturation magnetization *J*_s_ (magnetic moment in units of Bohr magnetons per ZnO formula units) on the Fe concentration in ZnO nanograined polycrystals obtained by the “liquid ceramics” method.

The saturation magnetization depends nonmonotonically on the Fe concentration (Figure 3). It increases more than ten times upon increase of the Fe content from 0 to 0.1 atom %. The magnetization drops down on further increase in Fe concentration and becomes almost indistinguishable from the background at around 20 atom % Fe. Above a concentration of 20 atom % Fe the magnetization increases again and reaches a value of about 0.09 emu/g (0.022 μ_B_/f.u.ZnO) at 40 atom % Fe.

## Discussion

**Figure 4:**
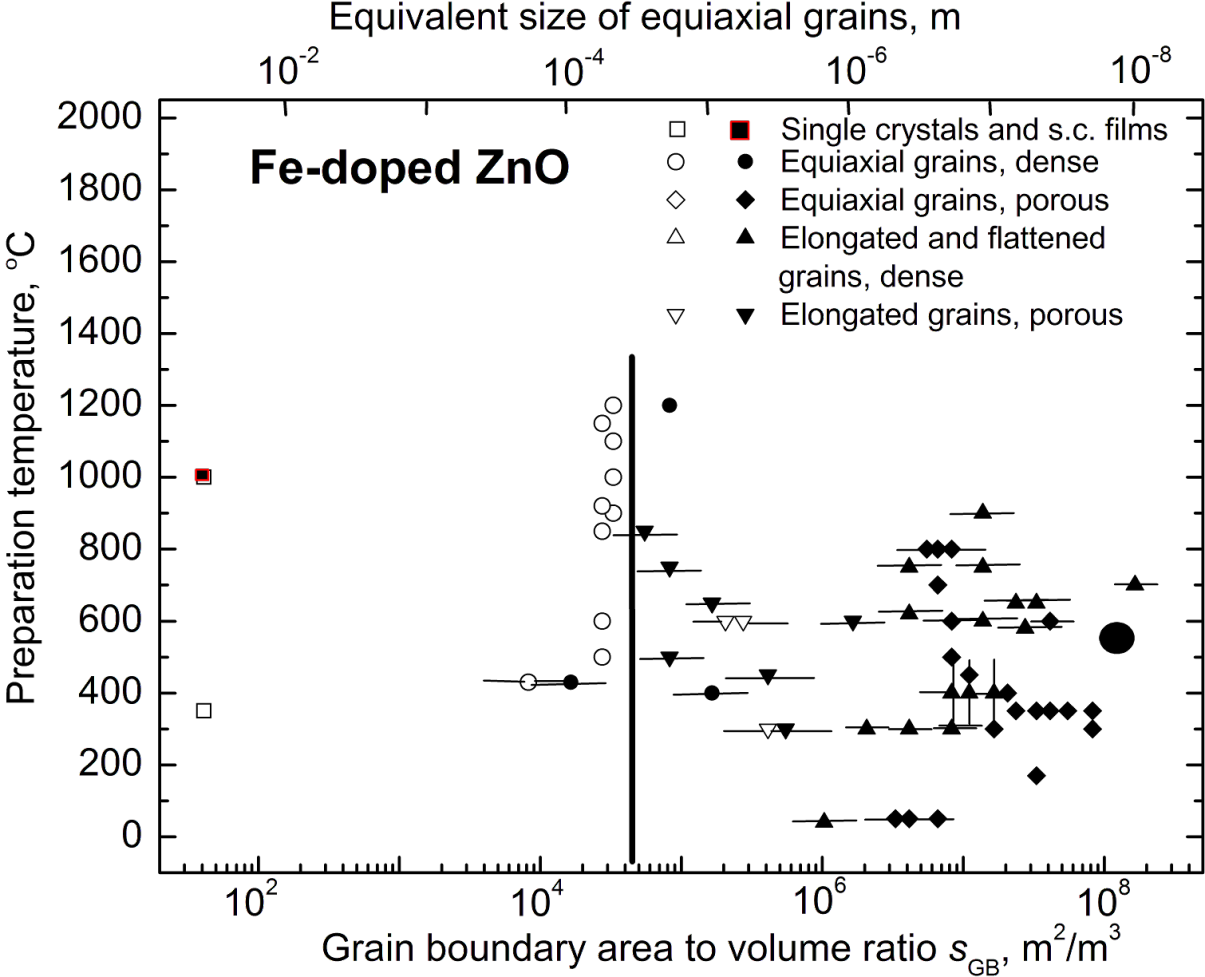
FM (full symbols) and para- or diamagnetic (open symbols) behaviour of Fe-doped ZnO in dependence on the specific GB area, *s*_GB_, the ratio of GB area to volume, at different preparation temperatures *T*. A vertical line marks the estimated threshold value *s*_th_. The enlarged symbol indicates the experimental data obtained by the authors’ own investigations (for symbols and references see the text).

We critically analysed the published papers on the search for possible ferromagnetic behaviour in the Fe-doped ZnO [22–66]. The results are summarized in Figure 4 in a *T*–*s*_GB_ plot (here *T* represents the annealing or synthesis temperature). They can be divided into three groups, depending on the *s*_GB_ value. First, the samples obtained by the magnetron and ion-beam sputter deposition or pulsed laser deposition (PLD) having small and very small grains are almost always ferromagnetic [22–47]. The respective (filled) points are grouping in the right part of the diagram in Figure 4. Second, the coarse-grained samples synthesised by the conventional powder sintering method, bulk single crystals or single-crystalline films are always diamagnetic or paramagnetic [48–55]. They are positioned in the left part of the diagram in Figure 4. In between one finds the third group of the data, namely obtained for the samples produced by chemical vapour deposition (CVD), solution combustion or wet chemistry methods. They have intermediate properties and can be either paramagnetic or FM [56–66].

We used different approaches in order to determine the value *s*_GB_, the ratio of grain boundary area to volume, basing on the published data [22–66]. Quite frequently the grain size has been carefully measured in published works (using TEM micrographs or XRD peak broadening) and directly quoted in the text. For other works we estimated the grain size ourselves basing again on the published TEM micrographs or XRD spectra. In such cases the points in Figure 4 have substantial error bars. The single crystals and single crystalline films [26,49] have no GBs, and formally the *s*_GB_ value is zero for them. We choose the value of *s*_GB_ = 4 × 10^2^ m^2^/m^3^ in order to indicate such data in Figure 4 (open squares). If the samples studied in the literature were poreless and contain equiaxial grains with mean grain size *D*, we calculated the *s*_GB_ as *s*_GB_ = 1.65/*D.* This formula is true for the space optimally filled with tetrakaidecahedrons (i.e., polyhedrons with 14 faces) [6]. It was used for the samples obtained by sintering of conventional [30,37,44,48–52,54–55,58,64] or nanopowders [24,29,32,41–42,46,53,56,59,65–66], or for films obtained by sol–gel method, pyrolysis, CVD or PLD [22–23,25,27–28,45]. If the samples mentioned in the analysed papers were not poreless, such as in the partly sintered powders (open and filled diamonds) [29,32,37,44,50,52–53,56,59,62,65], nanorods, or nanowires (open and filled down-triangles) [34,43,57,61], we introduced the additional porosity coefficient, *p*, for the *s*_GB_. *p* varies from 0 for nonsintered powders to 1 for the fully compacted polycrystals. We estimated *p* values using the published micrographs. In many cases the samples were poreless; however, the grains were not equiaxial but elongated [31,33,38–40,60] or flattened [35–36,45,47,66] (open and filled up-triangles). In these cases *s*_GB_ = 1.65*a*/*D*, *D* is the mean grain width and *a* is the aspect ratio (ratio of grain width to grain height). For the flattened grains *a* > 1, for the elongated ones *a* < 1.

The results for Fe-doped ZnO are summarized in Figure 4 in a *T*-*s*_GB_ plot. Indeed, the results clearly reveal a dependence of the FM behaviour on *s*_GB_. The samples are FM only for a certain threshold value *s*_th_. For the Fe-doped ZnO *s*_th_ = 5 × 10^4^ m^2^/m^3^. For pure ZnO *s*_th_ = 5.3 × 10^7^ m^2^/m^3^ [6], for Mn-doped ZnO *s*_th_ = 2.4 × 10^5^ m^2^/m^3^ [6], and for Co-doped ZnO *s*_th_ = 1.5 × 10^6^ m^2^/m^3^ [18]. This means that the addition of “magnetic” TM atoms to the pure ZnO did indeed drastically improve the FM properties of pure ZnO, as originally supposed in [1]. Moreover, Fe improved the FM properties of pure zinc oxide more effectively than Co and Mn. For the transition from paramagnetic to FM behaviour in the Fe-doped ZnO one needs many times fewer GBs than in the Co- and Mn-doped ZnO. The reason for the *s*_th_ difference for the pure ZnO and ZnO doped by Fe, Mn and Co could be also the strong segregation of Fe, Mn and Co in ZnO GBs. According to the estimations made in [4–5], the GB concentration of Mn or Co in the ferromagnetic nanograined samples can be several times higher than in the bulk. Our samples (large filled circle in the right part of the Figure 4) have very fine grains (10–30 nm). The grains are almost the smallest among the Fe-doped ZnO samples reported in the literature [22–66]. This means that the minimum in the concentration dependence *J*_s_(*c*_Fe_) (Figure 3) or the minima in the equivalent dependences *J*_s_(*c*_Co_) [18] and *J*_s_(*c*_Mn_) [17] for Co- and Mn-doped ZnO cannot be attributed to the fact that the *s*_GB_ value is larger than *s*_th_. On the other hand, it follows from Figure 3 that the *s*_th_ value could be different for different Fe concentrations. In other words, *s*_th_ = *s*_th_(*c*_Fe_) is generally concentration-dependent. For example for pure ZnO *s*_th_ = 5.3 × 10^7^ m^2^/m^3^. This means that the value *s*_th_ = 5 × 10^4^ m^2^/m^3^ (Figure 4) should be considered actually as the minimum possible one for the iron-doped ZnO.

In Figure 4 only the synthesis temperature and specific area of GBs are taken into account. However, the saturation magnetization *J*_s_ of the doped ZnO depends on the dopant concentration in a nontrivial manner (see for example [17]). In the case of Co-doped ZnO we also observed a strong increase of *J*_s_ for small amounts of Co added to pure ZnO [18]. The saturation magnetization decreased again above 5 atom % Fe. The presence of only one maximum in Co-doped ZnO [18] in comparison with Mn-doped ZnO [17] can be explained by the fact that the valence of Mn-ions changes from +2 to +3 and +4 with increasing Mn content and in the Co-doped ZnO Co always remains trivalent. Fe in ZnO can also change the valence from +2 to +3 [42,47,64–65]. Indeed, we observe a similar strong increase of *J*_s_ for small amounts of Fe added to pure ZnO (Figure 3). *J*_s_ increases more than ten times by the increase of Fe content from 0 to 0.1 atom %. The magnetization drops down at further increase in Fe concentration and becomes almost indistinguishable from the background at around 20 atom % Fe. The magnetization increases again above 20 atom % Fe and reaches a value above 0.09 emu/g (0.022 μ_B_/f.u.ZnO) at 40 atom % Fe. However, we do not observe a second drop of *J*_s_ with increasing Fe content (as took place in Mn-doped ZnO). This means that the concentration dependence of *J*_s_ in Fe-doped ZnO (Fe can be either di- or trivalent) is indeed, as we supposed in the Introduction, in a certain sense intermediate between the dependences for Co- (always trivalent) and Mn-doped ZnO (the valence of Mn-ions changes from +2 to +3 and +4 with increasing Mn content).

A strong increase of *J*_s_ by the addition of the first portions of “magnetic” TM atoms to the pure ZnO appears to be a general phenomenon, as reported in [17–18] and this work. At least, we observed it in all three cases of Mn-, Co- and Fe-doping. This means that the arguments of seminal work [1] are quite reasonable. However, the important difference is that Dietl et al. [1] predicted the transition to TM behaviour in bulk ZnO and, as we can see from the Figure 4 and respective plots in [6] and [18], the bulk ZnO (single crystals or coarse-grained polycrystals) remains non-FM even after the strong doping. The presence of grain boundaries is critically important for the FM behaviour of the zinc oxide. Moreover, it is specifically the grain boundaries and not the free surfaces that are crucial for FM behaviour. For example, it has been observed that the nonsintered ZnO nanoparticles doped with 16 atom % Co obtained by forced hydrolysis were not ferromagnetic [67]. This was despite the fact that their grain size of 40 nm was well below the threshold value of 1 μm for the Co-doped ZnO [18]. However, the same powder becomes FM after annealing at 400 °C. The TEM investigations revealed that the annealing leads to the partial sintering of nanoparticles [67]. In other words, the annealing formed the grain boundaries and they, in turn, caused the ferromagnetism.

In [4–5] we compared the adsorption of Co and Mn in GBs and at free surfaces of zinc oxide. It has been observed that the presence of GBs and free surfaces drastically increases the total solubility of Co and Mn in ZnO. For example, the second bulk phase (Co_2_O_3_ or Mn_3_O_4_) appears at 550 °C in single-crystalline or coarse-grained ZnO if the concentration of Co exceeds 2 atom % [4] and that of Mn exceeds 12 atom % [5]. In fine-grained poreless ZnO films (*D* < 20 nm) the total solubility of Co at 550 °C exceeds 33 atom % [4] and that of Mn exceeds 30 atom % [5]. In the fine-grained (*D* < 20 nm) powders only free surfaces and almost no GBs are present. The solubility of Co and Mn in such powders also increases but to a much lower extent (up to about 8 atom % Co and 20 atom % Mn) [4–5]. Similar investigations of the grain size influence on the total solubility of Fe-doped ZnO are now in progress; they give comparable results and will be published elsewhere. Simple calculations performed in [4–5] showed that the drastic increase of the total solubility of Co and Mn with decreasing grain size is due to the multilayer adsorption of dopants in GBs (up to 10 monolayers) and free surfaces (2–4 monolayers). From this point of view, the doped ZnO differs a lot from the metallic alloys where such a multilayer adsorption was not observed and the grain-size effect on the total solubility is much weaker [68–70]. Moreover, it has been observed in the Cu–Bi alloys that the Bi segregation in free surfaces is much stronger than that in GBs [70]. Therefore, it seems that the internal porosity in pure and doped ZnO cannot bring a significant input into FM behaviour.

The drop of *J*_s_ at a few percent of Co, Mn or Fe also seems to be a general feature of ZnO doped by the “magnetic” TM atoms. We supposed in our first paper on Mn-doped ZnO that the first minimum in the *J*_s_(*c*_Mn_) dependence is caused by the valence change from Mn^2+^ to Mn^3+^ and further to Mn^4+^ [17]. However, later we observed that *J*_s_ in Co-doped ZnO also drops down between 10 and 15 atom % Co after it reached a maximum at 1.2 atom % Co [18]. This happens despite of the fact that the valence of Co ions in ZnO is constant at Co^2+^. This means that the reason for the “first decrease” of *J*_s_ at a few per cent of “magnetic” TM atoms is not the valence change with increasing *c*_TM_. Most probably, the change of valence is responsible for the *J*_s_(*c*_TM_) behaviour at higher concentrations of TM atoms (above 10 atom % TM). As we can see, Mn has three different valence states (Mn^2+^, Mn^3+^ and Mn^4+^) and the respective *J*_s_(*c*_Mn_) curve has three minima and two maxima [17]. Fe has two different valence states (Fe^2+^ and Fe^3+^) and the respective *J*_s_(*c*_Fe_) curve has two minima and two maxima (Figure 4 in this work). The curve *J*_s_(*c*_Co_) for Co has only one maximum and two minima [18]. Therefore, we suppose that the “first minimum” between 1 atom % and 5–6 atom % of TM can be explained by the redistribution of doping atoms in the network of grain boundaries in TM-doped ZnO.

**Figure 5:**
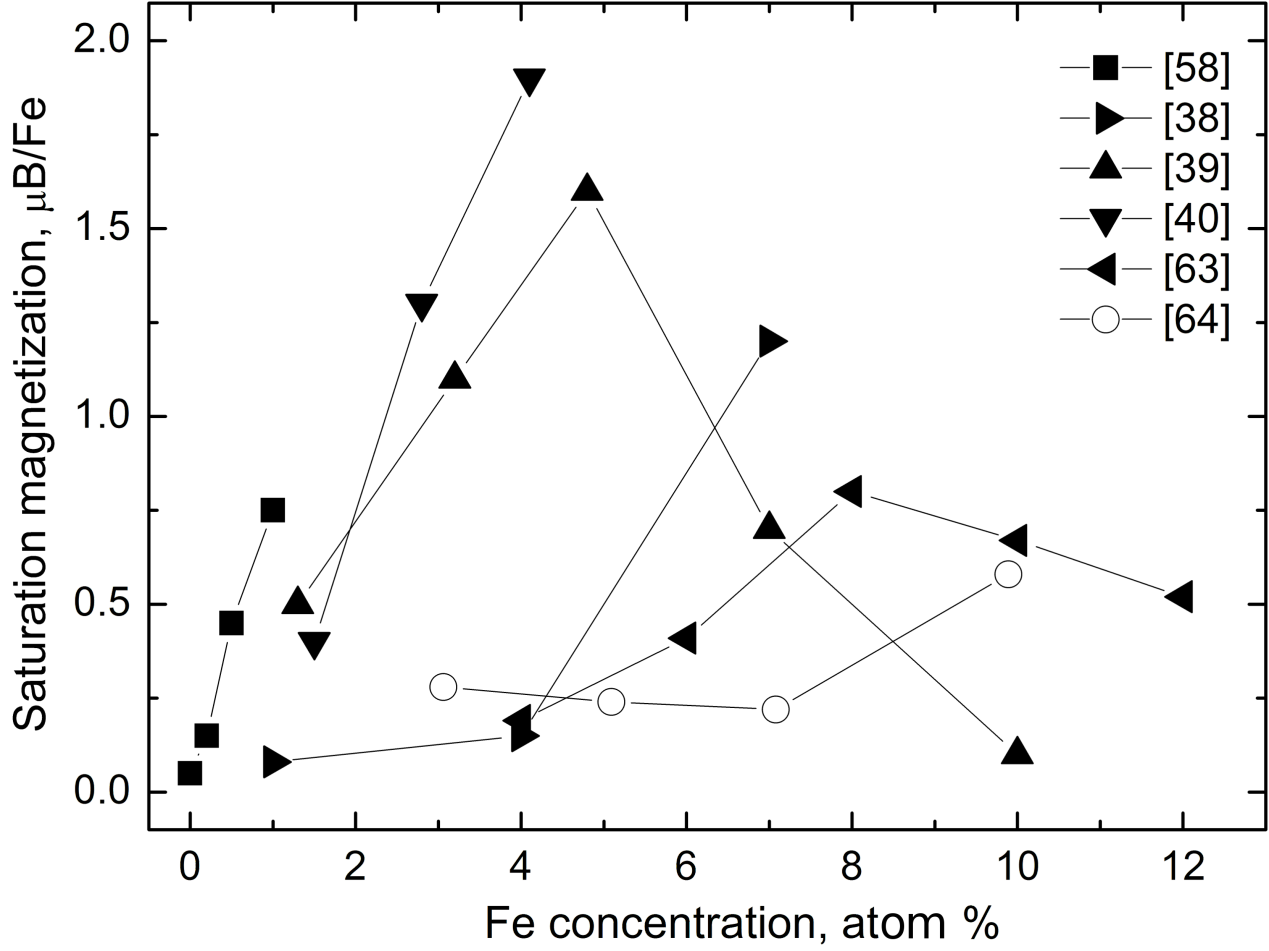
Dependence of the saturation magnetization (magnetic moment per iron atom in units of Bohr magnetons) on the Fe concentration in ZnO obtained by other methods such as magnetron sputtering (filled right- [38], up- [39], down- [40] and left-triangles [63]), solid-state reaction (filled squares [58]) and mechanical alloying (open circles [64]).

The nonmonotonic dependence of *J*_s_ on the Fe concentration has been observed in this work (Figure 3). A strong increase of *J*_s_ with the addition of small proportions of Fe atoms has been observed also in thin films with nanograined columnar structure deposited by magnetron sputtering (Figure 5, filled triangles) [38–40,63] and in samples synthesized by the conventional solid-state reaction having rather large (>10 μm) equiaxial grains [59]. If the ZnO films are deposited by magnetron sputtering, their *J*_s_ decreases above 5–8 atom % Fe with increasing iron content [39,63]. The ZnO samples obtained by mechanical alloying behave in a different way [64]. They demonstrate a very weak dependence of *J*_s_ on Fe content (see Figure 5, open circles).

The plot in Figure 4 and respective plots in [6,18] demonstrate that the presence of a certain amount of GBs is needed to transform non-FM zinc oxide into a FM state. The comparison between Figure 3 and Figure 5 shows that not only the specific area of GBs but also their character distribution (i.e., the spectrum of GB misorientations and inclinations) influences the *J*_s_ value. We observed previously that the texture or the amount of intergranular amorphous phase in the nanograined pure ZnO films drastically influences the FM properties even at the same grain size [68–70]. The GB structure also changes with increasing dopant content [71]. Moreover, by varying the synthesis conditions one can tailor the thickness of the amorphous intergranular layer and, thus, increase or decrease the *J*_s_ value [70]. It is well known that GBs with different character possess different adsorption ability [72–73]. If the GB networks have different topology, the GBs having various adsorption ability will be connected with each other in a different way. For example, the ZnO samples synthesized by the liquid ceramics method possess the uniform, equiaxial grains without visible pores inside [6,17–18,72–74]. The films deposited by the magnetron sputtering are also poreless and have columnar grains aligned perpendicular to the substrate [31,33,38–40,63]. Such samples, as well as sintered powders with equiaxial grains [58], have at low *c*_Fe_ the most similar *J*_s_(*c*_TM_) dependences (Figure 5) to our samples (Figure 3). The same is true also for the Mn- and Co-doped ZnO films [17–18]. If the contiguity of the GB network becomes weaker, as for example, in the samples composed of equiaxed nanograined balls, which in turn are loosely packed with each other (see Refs 25–29 in [17]), the first maximum in the *J*_s_(*c*_TM_) dependence becomes lower in comparison with the second one [17]. By further decrease of contiguity, such as in poreless samples with flattened grains (see Refs 24, 26, 28, and 92 in [18]), the *J*_s_(*c*_TM_) dependences becomes “stretched” along the *c*_TM_ axis, and the first maximum becomes shifted from 1 to about 10 atom % Co [18]. The continuous increase of *J*_s_ with increasing Co content in samples obtained by autocombustion or partly sintered nanorods (Refs 94 and 101 in [18]) can be considered as further “stretching” of the generic dependence shown in Figure 3. In other words, if the contiguity of the GB network (of “FM foam”) becomes low, the “first maximum” is not reached even at *c*_TM_ = 20–25 atom % [18]. In the samples obtained by mechanical alloying the *J*_s_(*c*_Fe_) dependence is very weak [64].

Thus, if we compare the *J*_s_(*c*_TM_) dependences for the Co-, Mn- and Fe-doped ZnO films having a dense, poreless structure with equiaxial grains, on the one hand, with samples having lower contiguity of the GB network (porosity, flattened grains, etc.) on the other hand, we can suppose that there is a kind of “generic” *J*_s_(*c*_TM_) dependence. This can be observed in the poreless, dense samples with equiaxial grains. This “generic” *J*_s_(*c*_TM_) dependence becomes “stretched” in the *c*_TM_ direction if the contiguity of the GB network decreases. As a result, the first *J*_s_ maximum moves from 1 atom % to 5–10 atom % and then disappears above 20–25 atom %. As a result, in the samples with low contiguity of the GB network only a weak increase of *J*_s_ with increasing *c*_TM_ remains.

## Conclusion

The influence of the specific area of grain boundaries *s*_GB_ on the presence or absence of ferromagnetism in Fe-doped ZnO has been analysed based on a review of numerous research contributions from the literature on the origin of the ferromagnetic behaviour of Fe-doped ZnO. An empirical correlation has been found that the value of the specific grain boundary area *s*_GB_ is the controlling factor for such behaviour. The Fe-doped ZnO becomes ferromagnetic only if it contains enough GBs, i.e., if *s*_GB_ is higher than a certain threshold value *s*_th_ = 5 × 10^4^ m^2^/m^3^. It corresponds to the effective grain size of about 40 μm, assuming a full, dense material and equiaxial grains. The value of *s*_th_ = 5 × 10^4^ m^2^/m^3^ is lower than that for pure ZnO *s*_th_ = 5.3 × 10^7^ m^2^/m^3^, that for Mn-doped ZnO *s*_th_ = 2.4 × 10^5^ m^2^/m^3^ and that for Co-doped ZnO *s*_th_ = 1.5 × 10^6^ m^2^/m^3^. This means that the addition of “magnetic” TM atoms to the pure ZnO did indeed drastically improve the FM properties of pure ZnO. Moreover, Fe improved the FM properties of pure zinc oxide more effectively than Co and Mn. We experimentally investigated the magnetic properties of Fe-doped ZnO thin films. The Fe concentration varies from 0 to 40 atom %. The thin films were deposited by using the wet-chemistry “liquid ceramics” method onto a sapphire substrate. The dense nanograined samples demonstrate ferromagnetic behaviour with *J*_s_ up to 0.10 emu/g (0.025 μ_B_/f.u.ZnO) and coercivity *H*_c_ ≈ 0.03 T. Saturation magnetisation depends nonmonotonically on the Fe concentration. It increases more than tenfold by the increase of Fe content from 0 to 0.1 atom %. The magnetization drops down at a further increase in Fe concentration and becomes almost indistinguishable from the background at around 20 atom % Fe. Above 20 atom % Fe the magnetization increases again and reaches a value of about 0.09 emu/g (0.022 μ_B_/f.u.ZnO) at 40 atom % Fe. In other published papers similar nonmonotonous dependences were observed in nanostructured films with elongated grains deposited by magnetron sputtering. These differences can be explained by the changes in the structure and contiguity of a ferromagnetic “grain boundary foam” responsible for the magnetic properties of pure and doped ZnO.
